# Intestinal factors promoting the development of RORγt^+^ cells and oral tolerance

**DOI:** 10.3389/fimmu.2023.1294292

**Published:** 2023-10-23

**Authors:** Rosina López-Fandiño, Elena Molina, Daniel Lozano-Ojalvo

**Affiliations:** Instituto de Investigación en Ciencias de la Alimentación (CIAL), CSIC-UAM, Madrid, Spain

**Keywords:** food allergy, oral tolerance, RORγt^+^ cells, regulatory T cells, ILC3s, retinoic acid, microbiome, dietary antigens

## Abstract

The gastrointestinal tract has to harmonize the two seemingly opposite functions of fulfilling nutritional needs and avoiding the entry of pathogens, toxins and agents that can cause physical damage. This balance requires a constant adjustment of absorptive and defending functions by sensing environmental changes or noxious substances and initiating adaptive or protective mechanisms against them through a complex network of receptors integrated with the central nervous system that communicate with cells of the innate and adaptive immune system. Effective homeostatic processes at barrier sites take the responsibility for oral tolerance, which protects from adverse reactions to food that cause allergic diseases. During a very specific time interval in early life, the establishment of a stable microbiota in the large intestine is sufficient to prevent pathological events in adulthood towards a much larger bacterial community and provide tolerance towards diverse food antigens encountered later in life. The beneficial effects of the microbiome are mainly exerted by innate and adaptive cells that express the transcription factor RORγt, in whose generation, mediated by different bacterial metabolites, retinoic acid signalling plays a predominant role. In addition, recent investigations indicate that food antigens also contribute, analogously to microbial-derived signals, to educating innate immune cells and instructing the development and function of RORγt^+^ cells in the small intestine, complementing and expanding the tolerogenic effect of the microbiome in the colon. This review addresses the mechanisms through which microbiota-produced metabolites and dietary antigens maintain intestinal homeostasis, highlighting the complementarity and redundancy between their functions.

## 1 Introduction

Food allergy is a prevalent disorder that has rapidly increased in the last 30 years, particularly in Westernized, developed societies (1). In its most common manifestation, it is an exacerbated type 2 innate and adaptive response to innocuous dietary antigens that eventually leads to IgE-mediated reactions to food components eliciting the release of inflammatory substances from basophils and mast cells (2, 3). Genetic features that cause deficient barrier functions or alterations in genes involved in Th2 responses are among the risk factors for allergy development (4), but they cannot explain its exponential increase, that rather points at environmental factors (5–7). Alterations in the intestinal microbiota or dysbiosis is central to most of these factors, a notion that expands and complements the hygiene hypothesis, originally postulated by Strachan in 1989 (8).

Oral tolerance, which is considered the default, homeostatic state whose failure leads to food allergy, is an active process of immune unresponsiveness to orally ingested antigens that protects from adverse reactions to food, locally in the gut but also systemically (9–13). Mechanistic studies in animal models of food allergy have provided abundant data supporting that tolerance is the outcome of a regulatory response, rather than the result of immune ignorance resulting from anergy, which designates T cell unresponsiveness to the antigen, or deletion, which denotes apoptosis of antigen-specific T cells (14–18). In this respect, induced regulatory T cells (Tregs) are considered essential for establishing peripheral tolerance to foreign antigens (19–21). Induced Tregs are complementary to thymus-derived Tregs, whose T cell receptor (TCR) repertoire is biased towards autoantigen recognition to maintain self-tolerance, although, according to some studies, the TCR repertoire of colonic thymic Tregs is sufficiently broad to recognize intestinal antigens and to maintain tolerance to them (22). Furthermore, it has been claimed that, when peripheral induction of Tregs is hampered, thymus-derived Tregs compensate to maintain the total intestinal Treg numbers and strengthen homeostatic capabilities (23, 24). Nevertheless, it is generally recognized that induced Tregs represent the majority of intestinal Tregs (25, 26). In the allergic state, induced Tregs are specialized in exerting the distinct functional role of controlling Th2 inflammation at mucosal surfaces through multiple suppressive mechanisms (19–21), while in the default tolerance state, it has been hypothesized that, at least in mice, their IL-2-sequestering action causes naïve CD4^+^ T cells to differentiate into a complex set of hyporesponsive T cells, lacking canonical T helper cell lineage markers and inflammatory functions, but having the potential to become peripheral Tregs themselves (27).

The establishment of a stable microbial community in the large intestine is a dynamic process that coincides with the maturation of the immune system and the generation of Tregs, responsible, not only for immune irresponsiveness to commensal microorganisms, but also for protection against food allergy (28–30). The intestinal mucosa, the main site of interaction between dietary antigens, the microbiota, and immune cells, is essential in the development and maintenance of tolerance, as it constitutes a distinctive environment that promotes the development and function of induced Tregs. This review addresses the mechanisms through which microbiota-produced metabolites and dietary antigens maintain homeostasis, highlighting the complementarity and redundancy between their functions and paying particular attention to recent reports that uncover new processes that mediate immune protection at barrier sites.

## 2 Intestinal microbiome and allergic disease

Several reports have demonstrated that the intestinal microbiota of children with food allergy is different from that of their non-allergic counterparts (31–33). Studies in animals have also allowed stablishing associations between an intestinal microbiota imbalance and food allergy. In the cholera toxin mouse model of sensitization to food proteins, the development of an allergic status parallels microbial alterations and the re-establishment of tolerance regenerates a healthy microbiota (34). Mice genetically susceptible to develop food allergy have a characteristic microbiota that transmits this susceptibility when transferred to germ-free mice (14) and, conversely, faeces from healthy infants protect mice from allergy induced by mucosal adjuvants (32, 33). Furthermore, dysbiosis in food allergy is also associated with high Th2 and IgE responses to commensal bacteria, showing an extensive breakdown of oral tolerance that goes beyond sensitization to food antigens (32). However, it has not yet been determined whether dysbiosis precedes and plays a role in the pathogenesis of allergy, or whether it is a consequence of the allergic process itself.

Microbiota maturation in humans is characterized by a high alpha diversity and dominance of genera within the Firmicutes phyla, to which commensal *Clostridiales* belong (35), and numerous reports point at *Clostridiales* as beneficial in the avoidance of food allergy. A Clostridia-containing microbiota is protective towards pathogen infections (36). It also stimulates the intestinal production of IL-22 by RORγt^+^ cells (37), that comprises ILC3s (a group of innate lymphoid cells that include LTi cells, involved in the development of lymph nodes, Peyer’s Patches and isolated lymphoid follicles) and T cells (γδT, iNKT and Th17 cells) (38). IL-22 targets intestinal epithelial cells (IECs) stimulating the production of antimicrobial peptides (AMPs) and mucus and helping to maintain and repair barrier integrity, which may reduce allergen uptake (39). However, the induction of factors that contribute to barrier function by Clostridia colonization does not provide full protection against food allergy (37). This effect rather arises from the ability of *Clostridiales* to increase in the number and function of colonic Tregs, as demonstrated in rodents colonized with these microorganisms (25, 26, 32, 33, 37, 40, 41). However, the observation that the generation of Tregs in mice occurs during a very specific time interval in early life, which coincides with weaning, and that it is sufficient to prevent pathological events in adulthood towards a much larger bacterial community and to provide tolerance towards diverse food antigens encountered later in life, points at a complex mechanism in which particular bacteria or Treg antigen-specificity may not be the determining factors (42–44). Thus, whereas there is a consensus that appropriate intestinal microbial stimuli during early life are critical for inducing a protective immunoregulatory network, it is controversial whether this stems from a sufficient level of microbial diversity (45–47) or from defined immunomodulatory bacterial species (32).

### 2.1 Foxp3^+^RORγt^+^ Tregs

*Clostridiales* are renowned for their ability to promote a tolerogenic environment through the induction of a particular subset of Tregs bearing the transcription factor RORɣt, which constitutes the major subset of colonic Tregs in the mouse and has also been found in human colon and peripheral blood (48–53). Foxp3^+^RORγt^+^ Tregs can also be generated by a specific, but wide, diversity of other bacteria (46, 53). These cells were proved to be functionally suppressive *in vitro* and *in vivo* (48, 51, 53, 54). In mice, colonic Tregs expressing RORɣt inhibit Th2 responses, avoiding the production of IL-4 and IgE (52). The involvement of Foxp3^+^RORγt^+^ Tregs in the mediation of oral tolerance in food allergy is further sustained by the finding that their frequency is reduced within peripheral blood mononuclear cells of allergic human patients and mice, despite normal frequencies of circulating RORɣt^+^ effector T cells, and by the enhanced susceptibility of mice with depleted *Rorc* (which encodes RORγt) expression to suffer vigorous anaphylactic responses (32). These findings open new perspectives for reinforcing oral tolerance through bacteriotherapy or faecal microbiota transplants (55). Interestingly, the function of Foxp3^+^RORγt^+^ Tregs is not restricted to intestinal health, since it has been recently found that colonic RORγt^+^ Tregs accumulate in injured muscles, under the influence of local inflammatory mediators, where they promote tissue regeneration through the control of local IL-17 production (56).

Despite the opposing primary regulatory and proinflammatory roles initially attributed to Foxp3 and RORγt, respectively the hallmark transcription factors of Tregs and Th17 cells, CD4^+^T cells that co-express Foxp3 and RORγt and produce IL-17 (albeit at lower levels than proinflammatory Th17 cells) constitute a distinct, stable cell lineage, rather than an intermediate subset during Treg and Th17 cell differentiation (48–50, 53, 54, 57). Moreover, recent studies indicate that Foxp3^+^RORγt^+^ Tregs are developmentally and functionally closely related to a homeostatic type of Th17 cells (58). The existence of protective Th17 cells responsible for the maintenance of epithelial barrier integrity underscores that RORγt and IL-17 are not *per se* determinants of pathogenicity, that rather depends on the *in vivo* environment and the presence of specific factors (59, 60). Whereas Foxp3^+^RORγt^+^ Tregs are regarded as induced Tregs, by virtue of the lack of expression of Helios or Neuropilin-1 (52), it has been claimed that thymus-derived Tregs can also acquire RORγt expression, particularly under proinflammatory conditions (61).

**Figure 1:**
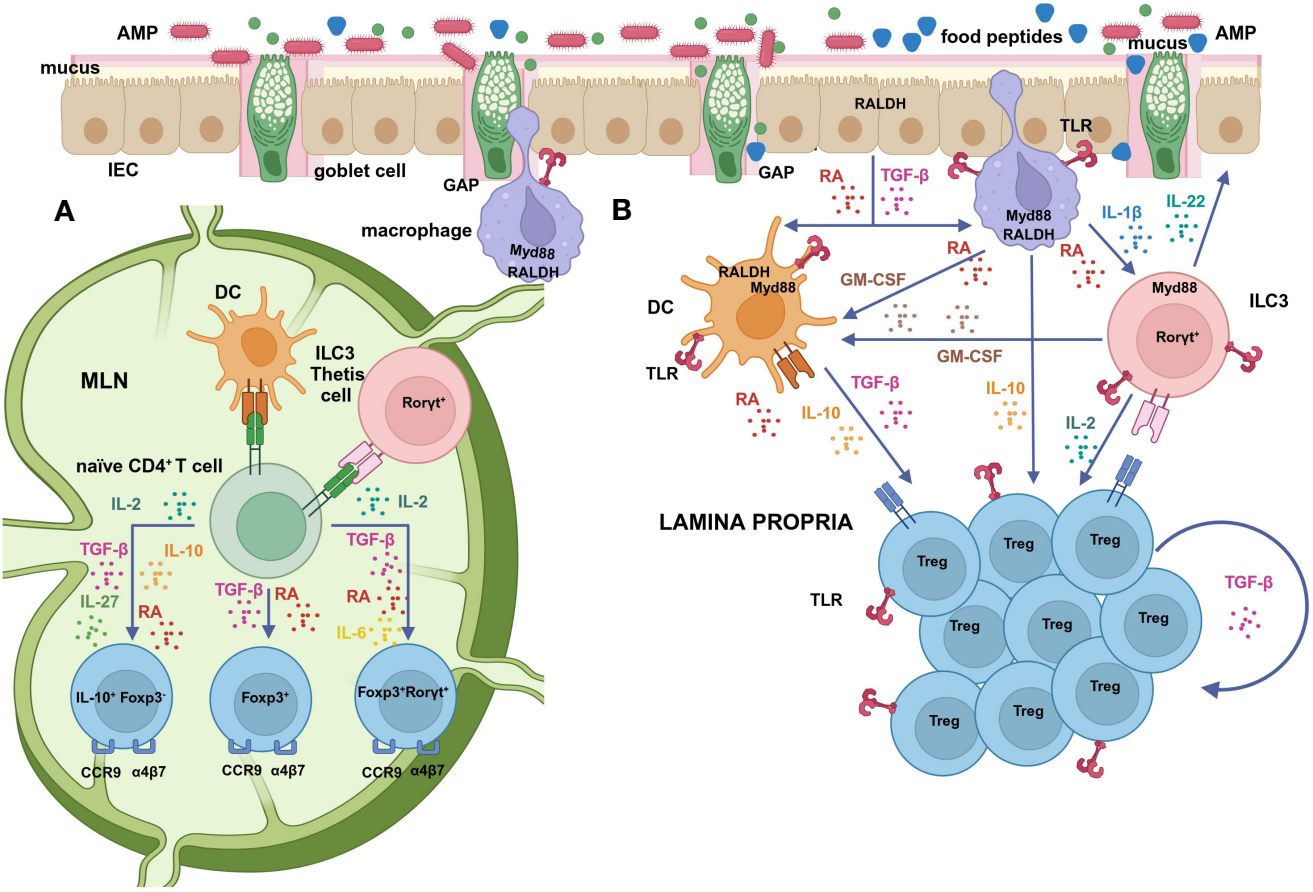
**(A)** Mucosal DCs, that take up antigens in the lamina propria and migrate to the MLNs to activate naïve T cells, are functionally specialized in the differentiation of Tregs by virtue of their ability to produce high levels of active TGF-β and RA (62, 63). RA facilitates Foxp3^+^ Treg generation, induced by microbial and food antigens uptaken via GAPs, through several direct and indirect mechanisms (64–66). The relative proportions of TGF-β, RA, IL-10 and IL-27 contribute to the induction of Foxp3^-^IL-10^+^, Foxp3^+^or Foxp3^+^RORγt^+^ Tregs that exhibit different resistance to inflammation (67–69). RORγt-expressing APCs, such as ILC3s and/or Thetis cells specifically enforce the RORγt^+^ Treg population (70–72). RA also promotes the expression of intestinal homing receptors (α_4_β_7_ and CCR9) on Tregs upon activation (73). **(B)** Cells that express RALDH constitutively, such as IECs and lamina propria and MLN stromal cells, act as a primary supply of RA that promotes its own synthesis by DCs (74, 75). Mucin-2, secreted by goblet cells, also imprints in DCs the transcription and activation of RALDH and the secretion of IL-10 and TGF-β (76). GM-CSF, produced by macrophages under the stimulus of RA (75), as well as by RORγt^+^ cells, mainly ILC3s, induces RALDH activity and the release of TGF-β by DCs and IL-10 by macrophages (77). Macrophages sense microbial cues and release IL-1β that drives ILC3s to produce GM-CSF (77) and IL-2, which further contributes to Treg generation (78). Additional signals, such as IL-4 and TLR ligands also synergize with RA and GM-CSF in DC education (75, 79, 80). RA has a direct stimulatory effect on RORγt^+^ cells, favouring the secretion of IL-22 by ILC3s in response to IL-1β, which stimulates the production of AMPs and mucus and maintains and restores barrier integrity (81, 82). Once Tregs have migrated to the lamina propria, IL-10 released by macrophages and DCs promotes the expansion of Foxp3^+^, and Foxp3^-^IL-10^+^ cells (69) and sustains their own production of IL-10 (83, 84). TGF-β produced by Tregs themselves or by eosinophils is likewise important to drive the expansion of Foxp3^+^RORγt^+^ cells (85, 86). The proliferation and maintenance of Foxp3^+^RORγt^+^ cells in the lamina propria also depends on costimulatory signals and MHC-II-driven stimulation by APCs (24) and on TLR signalling on Tregs themselves (32, 87). Created with BioRender.com.

TGF-β regulates the differentiation of Foxp3^+^, RORγt^+^ or double positive cells depending on its local concentration and on the existence of a proinflammatory environment, in a tightly controlled balance that is critical for immune homeostasis (59) (**Figure 1A**). High local concentrations of TGF-β favour the induction of Foxp3, which restrains the proinflammatory RORγt function, unless the cytokines IL-6, IL-21 and IL-23 stimulate the development of non-homeostatic Th17 cells (48, 49). Nevertheless, mice deficient in IL-6 have been described to develop significantly less Foxp3^+^RORγt^+^ cells than their IL-6-sufficient counterparts (52). Interestingly, TGF-β produced by Tregs themselves, under the influence of immunomodulatory commensal bacteria, promotes RORγt expression in nascent Tregs by exerting a specific non-redundant role with respect to TGF-β produced by cells of the innate immunity or by non-immune cells (85). Furthermore, once Foxp3^+^RORγt^+^ Tregs have differentiated from naïve CD4^+^ T cells and have acquired gut homing properties in the mesenteric lymph nodes (MLNs), TGF-β produced by eosinophils residing in close contact with them in the intestinal lamina propria drives their recruitment and expansion in the affected tissue (86). The proliferation and maintenance of Foxp3^+^RORγt^+^ Tregs in the lamina propria also depends on costimulatory signals by antigen presenting cells (APCs), mainly through ICOS and CD28, and likely on continuous MHCII-driven stimulation (24, 88) (**Figure 1B**).

The vitamin A metabolite retinoic acid (RA) promotes the generation of Foxp3^+^ Tregs and Foxp3^+^RORγt^+^ Tregs over Th17 cells *in vivo* (52). The most prevalent form of RA (all-trans RA) binds to retinoic acid receptors (RARs), which form heterodimers with retinoid x receptors (RXRs), working as nuclear receptors or ligand inducible transcription factors through the recognition of specific sequences designated retinoic acid response elements (RAREs) in the regulatory regions of several target genes (89). In the presence of TGF-β, RA may facilitate Foxp3^+^ Treg cell generation through several direct and indirect, non-mutually exclusive, mechanisms (64, 90), while it promotes the expression of intestinal homing receptors, α_4_β_7_ and CCR9, on Tregs to place them in the tissue where they are most needed (73) (**Figure 1B**). RA has been reported to enhance TGF-β-signalling by increasing Smad3 expression and phosphorylation (91). Indirectly, RA drives the differentiation of a more stable and complete Treg cell lineage *in vitro*, as compared with TGF-β alone, by releasing the STAT3-mediated IL-6 inhibition via the blockade of the expression of the IL-6 receptor (65, 66). In addition, *in vitro* and *in vivo* experiments showed that RA can hinder cytokine production by memory (CD44^hi^) CD4^+^ T cells (IL-21, IFN-γ, and, mainly, IL-4) (64), interfering with the ability of these cytokines to inhibit TGF-β-induced Treg conversion (92). In this respect, CCAAT/enhancer-binding protein (C/EBP), a family of transcription factors whose expression is upregulated by RA, was shown to ensure a stable induction of Tregs by conferring resistance towards inhibitory cytokines in both mouse and human systems (93). The observation that RA abrogates the suppressive activity of IL-4 on Foxp3 transcription underscores its role in favouring tolerance in Th2-mediated diseases (94). Furthermore, RA increases specific IgA responses at the expense of specific IgE responses in sensitized mice and decreases IgE production by human B cells (95). Nevertheless, RA, in *in vitro* experiments in the absence of TGF-β, rather than suppressing Th2 development, has been shown to enhance Th2 responses, what has been speculated to be a factor in sensitization to food allergens through non-oral routes (96).

Noteworthy, RA signalling has a direct effect on RORγt^+^ cells, since RA stimulation increases binding of RAR and RXR to the promoter region of the gene *Rorc*, thereby initiating its expression (81). In fact, the normal metabolism of RA by IECs, stromal cells and mucosal DCs has been identified as the stimulus that tilts the balance in favour of Foxp3^+^RORγt^+^ Tregs (38). Moreover, RA controls LTi cell maturation upstream of the transcription factor RORγt, which may explain why vitamin A deficiency in the foetus diminishes the size of secondary lymphoid organs (81). Similarly, RA/RAR signalling in RORγt is needed for a proper development of LTi cells and, in general, ILC3s in adult mice (82). RA inhibits IL-17A production by γδ T cells and enhances the production of IL-22 by γδ T cells and ILC3s *in vitro*, as well as *in vivo*, during intestinal inflammation in the mouse (97). Thus, vitamin A deficiency during early life hinders oral tolerance in mice, partially due to intestinal epithelium immaturity (98). As a result, absence of vitamin A in the adult promotes ILC2s, and likely allergic responses, as a tool to face barrier challenges deriving from less lymphoid tissue and reduced production of IgA and IL-22 (82, 99). As mentioned, IL-22 is important in tissue repair and maintenance of the mucosal barrier, but its production needs to be tightly regulated, because it is also highly expressed in several different chronic inflammatory conditions (38). Interestingly, RA also induces the production of IL-22 binding protein, a soluble inhibitory IL-22 receptor (IL-22R) that favours its inactivation, by lamina propria DCs in the small and large intestine of mice, as well as by human monocyte-derived immature DCs (100).

*In vitro*, most cells generated from naïve T cells using the classical Treg generating conditions simultaneously express the transcription factors Foxp3 and RORγt (48), suggesting that studies that have referred to Tregs induced TCR-triggering of naïve T cells with TGF-β and IL-2 as solely Foxp3^+^ cells did not discriminate between the two populations by appropriate staining for RORγt (101). Addition of IL-6, which opens the RORγt differentiation pathway, further drives co-expression of Foxp3 and RORγt (102), but this effect is transient and expression of Foxp3 subsequently declines (101). As in the *in vivo* situation, RA contributes to expand and stabilize the phenotype of double positive cells induced by TGF-β *in vitro* (48, 52, 101).

While RORγt expression in Tregs has been implicated in the maintenance of intestinal homeostasis, the mechanism through which these cells exert superior suppressive properties than Foxp3^+^RORγt^-^ Tregs is not yet clear (54). Foxp3^+^RORγt^+^ Tregs are characterized by a memory phenotype (CD44^hi^CD62L^lo^), with high levels of expression of *Icos* (ICOS), *Ctla4* (CTLA-4), *Il10* (IL-10) and *Irf4* (IRF4) (52, 54), as well as genes that confer specificity for mucosal tissues (54). In addition, they exhibit full demethylation of the Treg-specific demethylated region and substantial demethylation of other Treg-associated epigenetically-regulated genes, such as *Ctla4, Tnfrsf18* (GITR), and *Ikzf4* (Eos) and, therefore, possess a stable phenotype and functional specialization to fight inflammation at intestinal sites (54). The role of *Rorc* expression itself remains to be clarified, since inactivation of STAT3, as well as inactivation of c-Maf or impairment of TGF-β signalling, unlike inactivation of RORγt, have been shown to take part in the function of Foxp3^+^RORγt^+^ Tregs (103). IRF4 and STAT3, transcription factors critical for Th2 and Th17 cell differentiation respectively, are used by Tregs to specifically suppress Th2 and Th17 responses (104). For its part, the transcription factor c-Maf plays a critical role in the differentiation and activity of Foxp3^+^RORγt^+^ Tregs in the intestine, where it also helps to regulate the abundance of Th17 cells, induce IL-10 expression, and limit IgA responses to maintain the composition of the microbiota (105). TGF-β derived from Foxp3^+^RORγt^+^ Tregs was shown to restrain mast cell and IgE responses and, thus, diminish the susceptibility to food allergy (85). On the other hand, the transcription factor Blimp-1, preferentially expressed on Foxp3^+^RORγt^+^ Tregs, restrains the production of Th17 cytokines by these cells and maintains their suppressive function (106).

Of note, the TCR repertoire of Foxp3^+^RORγt^+^ Tregs is largely unique compared with other colonic T cell subsets, which suggests that TCR specificity may be sufficient to mediate their development (57). Analyses of the TCRs of colonic Tregs showed that this population is strongly shaped by antigens derived from commensal bacteria (25). Generation of these cells occurs mainly in the distal MLN, which drains the caecum and the proximal colon, likely after commensal antigen uptake via bacteria outer membrane vesicles or goblet cell-associated antigen passages (GAPs) (44, 107). These observations reinforce the concept that this Treg lineage is central for preventing excessive Th2 responses towards commensal bacteria that would otherwise compromise the integrity of the microbiota, in particular the niche of border-dwelling bacteria and their beneficial metabolic function (108).

CD103^+^ DCs cells, that take up antigens in the lamina propria and migrate to the MLNs to activate naïve T cells, have been proposed to be specialized in the initiation of Treg cell responses by virtue of their ability to produce high levels of active TGF-β and RA (62, 63) (**Figure 1A**). In particular, CD103^+^CD11b^-^ conventional type-1 DCs cells have been reported to display the highest retinaldehyde dehydrogenase (RALDH, the enzyme that oxidises retinal to RA) activity in the MLNs (109, 110). However, it was observed that both CD103^+^ and CD103^-^ DCs are redundant in promoting Treg differentiation (79, 111). Furthermore, under the influence of RA, that enhances actomyosin contractibility, lamina propria CD103^+^CD11b^+^ conventional type-2 DCs, with a mature proinflammatory phenotype, transmigrate into the intestinal epithelium to become immature intraepithelial DCs with tolerogenic properties further imprinted by RA itself and Mucin-2 (112). These observations point at a strong influence of environmental factors within the intestinal mucosa in DC education, but also suggest that other cell types may regulate Treg generation. Indeed, *in vivo* depletion of conventional DCs does not compromise Treg differentiation, implying the involvement of other APCs with pre-determined features (70). ILC3s are able to present antigens through MHCII to prevent the microbiota-dependent expansion of pro-inflammatory Th17 cells (113). Interestingly, deletion of MHCII in RORγt expressing cells resulted in failure to induce RORγt^+^ Tregs, calling into question the role of conventional DCs in this process (70–72). Recently, ILC3s were found necessary and sufficient to enforce the RORγt^+^ Treg population through a mechanism linked to an abundant expression of MHCII, CCR7-mediated migration to MLNs and activation of latent TGF-β (70, 71). Of note, a positive correlation between ILC3s and RORγt^+^ Tregs was also established in the human intestine (71). As mentioned, the efficient differentiation of RORγt^+^ ILC3s depends on RA (99), which also regulates their gut homing properties (114). However, another study proposed that ILC3 do not contribute to mucosal tolerance, but rather a novel lineage of RORγt^+^ APCs with a hybrid phenotype between DCs and thymic epithelial cells, which were termed Thetis cells (**Figure 1A**). Thetis cells are enriched in the MLN of mice at the specific time interval in early life that coincides with Treg differentiation, as well as in the MLN of human foetal samples (72). These studies reveal a sophisticated cross-regulation between RORγt-expressing cells in homeostasis that deserves further investigation.

### 2.2 Bacterial-derived triggers for Treg generation

The pathway underlying how specific intestinal commensal bacterial taxa stimulate the induction of colonic Foxp3^+^RORγt^+^ Tregs is thought to involve, in addition to microbial antigens and RA, bacterial fermentation products or metabolites, but has yet to be fully elucidated (52, 65, 115, 116). Short chain fatty acids (SCFAs) are thought to be required for their generation (40, 117, 118), although, according to some authors, there is no correlation between any SCFA and RORγt frequency, other Treg parameters or protection against food allergy, suggesting that they may play an indirect or subsidiary role (32, 53, 116).

The main mechanism by which SCFAs, mainly butyrate and to a lesser extent propionate, mediate their effects is histone deacetylase (HDAC) inhibition, that leads to epigenetic modification, loosened chromatin structure and activation of the expression of a wide range of genes (119). The involvement of G-protein coupled receptors (GPCRs), that sense SCFAs on the surface of IECs or immune cells is controversial, with reports indicating that the effects of SCFA are either dependent (120–122) or independent on their interaction with GPCRs (115, 117, 123, 124). Inhibitors of HDAC trigger RA-dependent transcriptional activation pathways. SCFAs, particularly butyrate, upregulate *Aldh1a1* (RALDH1) expression and increase RA conversion and active TGF-β production in human and mouse intestinal cell lines (115, 123, 124). Butyrate induces the expression of *Il10* and *Aldh1a2* (RALDH2) in murine colonic macrophages and DCs, enhancing their ability to promote the differentiation of naïve T cells into Foxp3^+^ and Foxp3^-^IL-10^+^ Tregs (121), and it also induces colonic Foxp3^+^RORγt^+^ Tregs in a DC-depending manner (52). In addition to indirect effects through APCs, SCFAs directly modulate mouse colonic Tregs, increasing their suppressive activity, proliferation capacity and *Foxp3* and *Il10* expression through HDAC inhibition (120). Butyrate enhances histone H3 acetylation in the *Foxp3* promoter and stabilizes *Foxp3* expression increasing its function (125). Of note, the positive action of SCFAs on epithelial barrier function and integrity through their effects on tight junction proteins and Mucin-2 secretion could also play an additional beneficial role in food allergy (126). Specifically, signalling through GPCR43 regulates colonic ILC3 proliferation and IL-22 production, which favours the expression of mucus-associated proteins and AMPs (127). Mucin-2, the main gel-forming mucin secreted by goblet cells, conditions IECs in mice to express tolerogenic factors, while it also increases the transcription and activation of *Aldh1a2* and the secretion of IL-10 and TGF-β by DCs (76).

Primary bile acids, generated from cholesterol in the liver and released in the intestine to aid nutrient absorption, are processed by the microbiota to generate secondary bile acids. Both act as signalling molecules on cells of the innate and adaptive immunity (128). Some secondary bile acids increase the induction of Foxp3^+^RORγt^+^ T cells *in vitro* by acting on DCs to decrease their immunostimulatory properties (129). In addition, specific bile acid metabolites enhance Treg differentiation *in vivo* through epigenetic modification that causes loose chromatin structure in the *Foxp3* gene (130, 131). Similarly, it was found that bile acids generated in response to diet and biotransformed by certain bacteria induce colonic Foxp3^+^RORγt^+^ T cells via their interaction with the vitamin D receptor (VDR), which is highly expressed in colonic IECs, DCs and Tregs (116). VDR and the principal receptor for bile acids, farnesoid X receptor (FXF), form heterodimers with RXRs or RARs to function as ligand-induced transcription factors in target genes, and interaction between nuclear signalling by vitamin D or bile acids and RA has been documented (132). Bile acids have been found to stimulate a RARE response in DCs via FXR-RAR interactions (133) and, conversely, RA has been reported to upregulate FXR pathway genes and to modulate hepatic homeostasis and lipid metabolism (134). In this regard, it would be interesting to determine the crosstalk between FXF, RARs and RXRs and whether their respective ligands can activate the others’ pathway to stimulate oral tolerance.

In addition to SCFAs and bile acids, indole-containing molecules, produced by the microbiota at the expense of dietary tryptophan, modulate the intestinal Treg compartment by the activation of aryl hydrocarbon receptors (AhRs) present in several immune cells, which act as ligand-activated transcription factors (135). Both the depletion of tryptophan from the environment and the increase in its metabolites polarize CD4^+^ T cells to a Treg phenotype (136). AhRs are key regulators in the development of RORγt^+^ ILC3s and Th17 cells and in IL-22 production, as well as in promoting Treg homing and function (137, 138). Moreover, AhRs are highly expressed in intestinal Tregs, helping to regulate Foxp3^+^RORγt^+^ Treg homeostasis (139, 140).

Additional factors from commensal bacteria may promote tolerance through classical innate immune response pathways. Toll Like Receptors (TLRs), which are the main receptors that recognize structurally conserved molecules derived from commensal bacteria, trigger immune responses by causing myeloid differentiation primary response gene 88 (MyD88) signalling. MyD88, which also works as an adaptor for the IL-1β receptor family, leads to a variety of functional outputs, including the activation of nuclear factor κB (NF-κB) that works as a central node of inflammatory pathways (141). Whereas the contribution of conserved bacterial molecular patterns in the generation of Foxp3^+^ Tregs and Foxp3^+^RORγt^+^ Tregs was discarded in experiments with MyD88-deficient mice (26, 52), other studies have recognized the role of the TLR/MyD88 axis in promoting Treg formation. MyD88 signalling contributes to DC education, as mucosal DCs from MyD88-deficient mice express low levels of RALDH enzymes (80). Thus, cell surface polysaccharides can promote Treg generation in the intestine through the induction of TGF-β from lamina propria DCs via TLR2, as described for *Clostridium butyricum* and *Bifidobacterium bifidum* (142, 143). TLR ligands educate *Aldh1a2* expression and RALDH activity in APCs (74, 79, 111, 144). Furthermore, TLR/MyD88 sensing of the commensal microbiota by intestinal macrophages elicits the production of IL-1β, which activates ILC3s to produce granulocyte-macrophage colony stimulating factor (GM-CSF) that further promotes *Aldh1a2* expression in intestinal DCs (77), as well as IL-2, which contributes to maintain Treg cells and oral tolerance (78) (**Figure 1B**). In addition, the microbiota can provide TLR signals to CD4^+^ T cells in the absence of APCs. Thus, the molecule polysaccharide A (PSA) from *Bacteroides fragilis* induces Tregs that produce IL-10 and TGF-β by signalling directly on CD4^+^ T cells through TLR2 (145). PSA also potently boosts Treg function through TLR2 signalling on CD4^+^Foxp3^+^ Tregs themselves (146). Enhancement of the expression of Foxp3 and, consequently, of the suppressive capacity of Tregs has been reported for agonists of TLR2, 4 and 5 (147), and RA further synergizes with TLR2 directly on T cells to increase the production of IL-10 (148). Direct sensing of bacteria by nascent Tregs induces RORγt^+^ expression, promotes IgA immunity and regulates the commensal flora avoiding dysbiosis by the MyD88 pathway (32, 87).

On the other hand, nociceptor neurons, important components of the intestinal nervous system, exhibit TLRs and respond to microbial stimulation to regulate inflammation (149). In this respect, the induction of colonic Foxp3^+^RORγt^+^ Tregs by selected microbiota correlates with the downregulation of neuronal transcripts, including *Il6* expression, both *in vitro* and *in vivo* (150). This indicates a functional connection between microbial signals delivered by Foxp3^+^RORγt^+^ Treg inducers and neuronal activity that results in diminished neuronal density and function, which, through IL-6, regulates Treg numbers and phenotype, including the RORγt proportion (151).

In Foxp3-deficient mice, unrestrained MyD88-driven proinflammatory signals lead to multisystem inflammatory diseases, emphasising that a critical function of Tregs is to control inflammation by inducing tolerance to stimuli from commensal bacteria (152). However, a paradox arises, as signalling through the common TLR adaptor MyD88 alerts the immune system to danger from pathogens, triggering Th1-driven inflammatory reactions through NF-κB, that can inhibit the suppressive capacity of Tregs (141). The positive effect of TLR activation on the differentiation of Tregs may be part of a negative feedback loop to restrain inflammation, as postulated for thymus-derived Tregs (153). Eventually, the outcome of MyD88 signalling in terms of Treg development is likely context-specific: under the influence of low levels of proinflammatory cytokines, it would contribute to the maintenance of homeostasis by actively promoting Tregs, while an excessive inflammatory environment would otherwise favour the development of Th1 or Th17 cells. This underlines the need for strict regulation of cytokine expression for homeostatic effects to be properly manifested. Interestingly, RA exerts an autocrine effect on DCs, inducing the negative regulator SOCS3, which acts as a negative feedback loop to suppress TLR-induced release of pro-inflammatory cytokines, which, otherwise, would antagonize Treg generation (154). Furthermore, given that commensal and pathogenic microorganisms share molecular patterns, the switch from tolerance to immunity was suggested to be provided by a microbiota-independent signal by CD4^+^ T cells through CD40L on tolerogenic DCs (155). This abrogates Foxp3^+^RORγt^+^ Treg induction and increases Th1 and Th17 cells when it is necessary to boost immune responses (155).

The finding that, in mice, the phenotype of colonic Foxp3^+^RORγt^+^ Tregs is strain-dependent, quantitatively induced by the mother during a very early and short window, stably maintained throughout adulthood and passed on for multiple generations, led Ramanan et al. to postulate that the influence of the microbiome was under the control of a non-microbial element. According to these authors, the maternal transfer of IgA through the milk leads, depending on its level, to a differential coating of intestinal bacteria, changing their stimulatory capacity to induce Foxp3^+^RORγt^+^ Tregs. In adulthood, IgA and Foxp3^+^RORγt^+^ Tregs regulate each other in a negative feedback loop, while the transfer of IgA and plasma cells to the mammary gland transmits the phenotype to the progeny (156).

## 3 Tolerogenic immune responses to dietary antigens

Whereas the colon harbours the highest density and abundance of antigens from the microbiota, the small intestine holds the greatest load of dietary antigens. In the small intestine, the site of food absorption, is where oral sensitization to dietary proteins and amplification of allergic reactions take place and, therefore, where tolerance to foods is mainly sustained (18). As compared with distal lymph nodes, the small intestine draining lymph nodes preferentially give rise to tolerogenic responses to food, by virtue of their differential stromal and APC gene signatures and compartmentalized absorption and drainage, which results in differential nutrient exposure (157). Foxp3^+^RORγt^+^ Tregs have been found in the small intestinal lamina propria of mice, where Foxp3 is expressed by 17-20% of CD4^+^ T cells (49) and, approximately, 68% of Foxp3^+^ cells express RORγt at steady-state versus 72% in the colonic lamina propria (52). Bacteria are more numerous in the ileum, where they can regulate allergic responses to dietary antigens. In particular, the presence in the ileum of the clostridial species *Anaerostipes caccae* was related with protection against food allergy (33). Nevertheless, it is unlikely that the bacteria that colonize the small intestine, much less abundant, exert direct effects and, without underestimating the role played by immune cells migrating from the colon to the MLNs or by metabolites or cytokines produced by bacteria in the large intestine, it is generally recognised that the induction of Tregs in the small intestine is basically independent of interactions with the microbiome (26).

As mentioned, Foxp3^+^RORγt^+^ Tregs that confer sustained tolerance emerge throughout a defined interval, during which dietary antigens are delivered to the colonic immune system. This developmental window opens at the time when the concentration of epidermal growth factor (EGF) present in breast milk undergoes a temporal decrement that allows goblet cells in the large intestine to form GAPs. GAPs favour the transfer of luminal antigens to the colonic lamina propria and the expansion of long-lived Foxp3^+^RORγt^+^ Tregs that promote long-term oral tolerance to commensal bacteria and food antigens, even those encountered for the first time later in life (42, 44, 158). Closure of colonic GAPs restricts the passage of luminal antigens, ending the developmental window for the expansion of Tregs, although the responsible factor is controversial (44). The microbiota has been reported to inhibit colonic GAP formation due to goblet cell sensing of bacterial molecular patterns, through TLRs, in adult mice (159). Thus, not only the superior functional features of Foxp3^+^RORγt^+^ T cells, but also the length of the period of time during which they expand, have been suggested to depend on the properties of the bacteria responsible for their generation (158). In the small intestine, however, due to the lower expression of TLRs in goblet cells, the microbiota does not inhibit GAP formation (159). This allows a continuous generation of Tregs after weaning, although these cells have a limited lifespan and undergo rapid turnover when animals are deprived of food antigens (88, 158). These observations may provide an explanation why continuous allergen exposure is required to maintain tolerance after a certain age and stimulate the investigation of the beneficial cues that promote the right conditions for antigen encounter.

Important questions are whether the tolerogenic effect of Foxp3^+^RORγt^+^ Tregs is allergen-specific and whether these cells depend on the microbiota for their generation. Indeed, Foxp3^+^RORγt^+^ Tregs specific to food antigens can be generated in the small intestine (52). Furthermore, a local microbiota-independent induction of these cells has been reported in the small intestine of germ-free mice despite depletion in the colon, showing that they do not differentiate exclusively under the influence of microbial signals (160). Foxp3^+^RORγt^+^ Tregs developed in the small intestine by dietary proteins in the absence of commensal microbiota suppress Th2-driven immune responses to food, although they are more transient than colonic Tregs (88). Another issue is whether it is possible to promote their stable generation later in life, through the induction of intestinal tolerogenic factors and the regulation of GAP formation and antigen delivery. In this regard, several studies have shown that Foxp3^+^RORγt^+^ Tregs continue to develop after weaning and that commensal bacteria can induce them in adult mice (32, 52, 53).

### 3.1 Hydrolysed food proteins and dietary epitopes

In mice, hydrolysed egg white proteins were shown to offer long lasting desensitization to egg allergy, more effectively than treatment with the intact allergen, through the upregulation of *Tfgb1* (TGF-β), *Il10*, *Il17* (IL-17), *Foxp3*, and *Rorc* in the small intestinal lamina propria, PPs, and MLNs, and the development of Foxp3^+^ cells that simultaneously expressed RORγt (34, 161). Furthermore, mice exhibited an enhanced expression of genes associated with epithelial integrity, such as *Il22* (IL-22) and *Il22ra2* (IL-22R), in the duodenum, and *Muc2* (Mucin-2) in the colon, as well as *Aldh1a1*, *Aldh1a2*, and *Csf2* (GM-CSF) in intestinal lymphoid and non-lymphoid tissues, indicating that food protein hydrolysates exert protective barrier functions and provide tolerizing signals to DCs via the activation of RALDH enzymes (34). While dietary antigens had been shown to induce the expression of RORγt and Foxp3 in naïve CD4^+^ T cells specific for that antigen transferred to mice (52, 88), no previous reports had shown the role of food peptides in the resolution of food allergy through the enhancement of vitamin A metabolism and the development of Tregs bearing the transcription factor RORγt. Remarkably, in humans, an association between successful immunotherapy against aeroallergens and the induction of genes involved in RA metabolic pathway has recently been observed (162).

*Ex vivo* studies confirmed that the hydrolysate upregulated *Aldh1a1* in IECs, which, in turn, conditioned DCs to overexpress *Aldh1a2* and *Tgfb1*, to release IL-6 and IL-10, and to promote the generation of TGF-β-secreting Foxp3^+^RORγt^+^ cells from CD4^+^ T cells (163). TLR stimulation is a possible mechanism through which food peptides enhance RALDH activity on murine DCs (164). Food protein hydrolysates can activate TLRs depending on peptide size and sequence (165), which may explain why intact proteins are less suited than hydrolysed proteins to generate Foxp3^+^RORγt^+^ Tregs, even after having undergone an *in vivo* digestion process (34). Thus, hydrolysates confer specific properties on DCs by means of the upregulation of tolerance-promoting mediators downstream of TRL signalling, such as *Aldh1a2*, *Tgfb1, Il10, Il27* (IL-27), *Il33 (IL-33)*, *Jag2* and *Dll4* (coding for the Notch ligands Jagged2 and Delta4 respectively) and *Tnfsf4* (OX40L) (164). IL-27 supports the differentiation of Foxp3^-^ Tregs and their IL-10 production (166). IL-33, released by DCs under the influence of the epithelial alarmin IL-33 itself as well as TLR ligands (167), promotes RA signalling in CD4^+^ T cells, enhancing TGF-β-mediated differentiation of Tregs and favouring their accumulation and maintenance (168). This mechanism likely represents a feedback loop by which alarmins limit inflammatory damage at barrier tissues. For its part, Notch signalling on CD4^+^ T cells, depending on the ligand, enhances Foxp3 expression *in vitro* and helps to maintain Tregs *in vivo* (169). In particular, the Notch family ligands Jagged1 and Jagged2 promote TGF-β signalling and *Foxp3* transcription, while concomitant OX40L-OX40 interaction delivers survival signs, allowing Treg expansion (170).

These effects are similar to those exerted by RA, since regulation of DCs through RA also requires MyD88, the adaptor protein conventionally associated with TLR signalling (74, 80). In addition, RA induces *Tlr1* (TLR1) and *Tlr2* (TLR2) mRNA in DCs and therefore contributes to sensitize DCs to TLR ligands (74). However, unlike RA (79), the hydrolysates significantly upregulate *Il10*, possibly because TLR signalling on DCs enhances IL-10 independently of the induction of RALDH activity (154, 171). Accordingly, DCs conditioned with the hydrolysate enhance Foxp3 expression in cocultured CD4^+^T cells and, in line with their high expression of *Il27* and *Il10*, they also increase the level of Foxp3^-^IL-10^+^ cells, depending on the concentration of exogenous TGF-β (164). Interestingly, a singular phenotype of Foxp3^-^IL-10^-^CD4^+^ T cells effective against Th2 responses in food allergy was claimed to be induced by DC differentiated in the presence of RA and exposed to TLR stimulation (67), illustrating that the modulation of RA, IL-10, and IL-27 levels by TLR ligands may modify the balance of Treg subsets with different properties (68, 69). IL-10 produced by TLR-stimulated DCs and macrophages plays additional homeostatic roles as it helps to expand Tregs in the lamina propria, once these cells have been generated and have acquired gut homing properties in the MLNs (172). Foxp3^+^ Tregs need to respond to IL-10 to sustain IL-10 production and restrain Th17 responses (83). Similarly, although IL-10 is not essential to induce Foxp3^-^IL-10^+^ Tregs *in vivo*, it is crucial to maintain the production of IL-10 and preserve their regulatory activity (84) (**Figure 1B**).

As indicated, signalling by commensal bacteria on TLRs expressed on Tregs may directly control immune responses in the absence of APCs. Consequently, incubation of CD4^+^ T cells from naïve mice in the presence of hydrolysate, without DCs, upregulated *Aldh1a1*, *Aldh1a2*, *Tgfb1*, and *Il6* expression and induced RALDH activity through TCR stimulation (164). Although information regarding RALDH expression and subsequent production of RA by CD4^+^ T cells is very scarce, it has been reported that allogenic, and likely other types of stimulation, increase RALDH activity in conventional CD4^+^ Tregs, and in particular in Tregs, delivering resistance to cytotoxic agents and immunological tolerance (173). Noteworthy, since nociceptor neurons express and can be triggered via TLRs, it is possible that they also sense food antigens in a way similar to how they sense signals from the microbiota (174).

Noteworthy, unlike the case of sensitized mice, the administration of hydrolysates to naïve mice does not modify the expression of enzymes involved in the generation of RA, which implies the absence of effects on vitamin A metabolism under homeostatic conditions (34). Accordingly, it was found that a Th2-skewed environment is favourable to the induction of a Foxp3^+^RORγt^+^ phenotype (163, 164). These observations are consistent with the positive effect that certain Th2 mediators, such as IL-4 or IL-13, exert, in synergy with RA, GM-CSF, and TLR ligands, on the activation of *Aldh1a2* expression and on the suppression of the production of pro-inflammatory cytokines by DCs, contributing to promote Tregs (75, 175). Other regulatory cells, albeit of innate origin, ILCregs, are not found at the steady state in nasal tissues of human subjects or lungs of mice, but rather during Th2 inflammation, because their transformation from ILC2s is promoted by the upregulation of RALDH enzymes in airway epithelial cells (176). These cells are induced during immunotherapy in humans in response to RA and mediate tolerance to aeroallergens, helping to restore epithelial integrity and suppress Th2 responses (162). Likewise, RA enhances the *in vitro* TGF-β-driven conversion of memory Th2 cells into Tregs that, once adoptively transferred, suppress proliferation and cytokine production by Th2 memory cells and allergen-specific IgE production (177).

It is well known that a Th2 environment has a role in impairing Treg-driven tolerance in allergic disease by restraining Treg generation and function (20) or by driving Treg reprogramming to a Th2 (178) or a Th17 (179) phenotype. However, IL-4 has also been reported to positively regulate Foxp3^+^ Treg stability and function in the course of Th2 inflammation processes *in vivo*. Tregs from IL-4-deficient mice exhibit decreased persistence and granzyme expression, indicating that IL-4 supports Treg suppressive capacities (180). Similarly, the absence of IL-4 receptor responsiveness on Foxp3^+^ Tregs exacerbates airway inflammation in asthmatic mice (181). Notably, C/EBP functions mainly in the presence of IL-4 and IFN-γ, conferring resistance to these inhibitory cytokines during Treg generation (93).

TLR ligands, together with other signals, such as TGF-β, condition DCs to express indoelamine 2,3-dioxygenase (IDO), an enzyme that catabolizes tryptophan into different metabolites, such as kynurenines, that activate AhRs (182). The IDO-driven immunomodulatory capacity can be extended between different murine and human DC subtypes. Thus, under the influence of TLR ligands, the subset of conventional type-1 DCs that expresses IDO can induce this ability in co-cultured non-tolerogenic conventional type-2 DCs by activating AhRs (whose expression is also stimulated by TLR-signalling) through the action of L-kynurenine (183). All together, these results show that food antigens may work, analogously to microbial driven signals to stimulate cells of the innate and adaptive immune system and promote mucosal tolerance at different levels.

Another possible mechanism of action of immunomodulatory peptides to counteract food allergy involves interference with the activation of formyl peptide receptors (FPRs). Among them, FPR3, expressed intracellularly in DCs and whose interaction with allergens polarizes naïve CD4^+^ T cells to a Th2 phenotype, can be antagonized by peptide ligands (184). Very recently, it was found that peptides derived from ovalbumin bind and activate caspases 3 and 7 in small intestinal IECs to release a non-lytic 13 kDa N-terminal fragment of Gasdermin D that induces the expression of MHCII and supports IEC antigen-presenting ability to convert naïve CD4^+^ T cells into Foxp3^-^IL-10^+^ cells (185). However, whereas there seems to be an optimal peptide length and sequence to induce tolerance through this mechanism, these aspects require further investigation.

β-hexosaminidase, a conserved enzyme among the Bacteroidetes phylum, as well as a minimal peptide epitope composed of 9 amino acids, were identified as bacterial antigens that drive the differentiation and regulatory function of Foxp3^–^CD8αα^+^CD4^+^ intraepithelial lymphocytes (CD4IELs), that work, together with Tregs, in the maintenance of intestinal homeostasis (186, 187). Interestingly, CD4IELs also depend on RA and TGF-β signalling for their development and can derive from Tregs that lose Foxp3 upon migration to the epithelium (188). Similarly, dietary proteins promote, by themselves, clonal selection and epithelial adaptation of CD4^+^ T and Tregs in the small intestine, a process that is further boosted by signals from the microbiota (18).

On the other hand, food peptides can influence intestinal homeostasis by reinforcing barrier function, supressing inflammatory responses and stimulating mucin-2 secretion from goblet cells (189). Moreover, the microbiota, in different locations of the intestine, is shaped by the availability of dietary protein and protein fragments, which may indirectly influence the mucus barrier (190). This suggests that, in addition to providing DC and CD4^+^ T cell education, peptides could also drive tolerogenic responses by helping to regulate GAP formation and healthy antigen delivery (191).

## 4 Concluding remarks

Intestinal mucosal immune responses that lead to oral tolerance depend on a multifaceted and sophisticated network of non-immune and immune mechanisms. We have tried to present the complexity involved in maintaining homeostasis in intestinal tissues, where the same actors play different roles in different contexts, as exemplified by factors that may mediate anti- or pro-inflammatory effects depending on whether they take part of a health or disease situation. The most active drivers of intestinal tolerance, Tregs, exhibit functional plasticity to adapt to specific environments and inflammatory conditions by acquiring different migratory and suppressive mechanisms to avoid uncontrolled inflammatory responses against food and commensal microbiota. Among Tregs, induced CD4^+^ T cells coexpressing Foxp3 and RORγt are crucial for the maintenance of homeostasis in the intestine, probably acting in cooperation with other Foxp3^+^ or Foxp3^-^ suppressive CD4^+^ T cells. The impact of Foxp3^+^RORγt^+^ Tregs extends beyond their site of generation, illustrating that issues such as the antigen specificity or Treg cross-reactivity are open questions.

In addition, in the intestinal lamina propria there are unique populations of immune cells also expressing the lineage-defining transcription factor RORγt, such as ILC3s, γδT, and Th17 cells, which exert passive tolerance roles through the enforcement of the mucosal barrier system, while RORγt^+^ APCs have been deemed responsible for the induction of RORγt-expressing Tregs. The functional interactions among RORγt^+^ cells in oral tolerance induction and allergy prevention or therapy, although mainly illustrated in mouse models, underscore the importance of strategies to promote their expansion. The microbiome induces the expression of colonic RORγt cells that are particularly well suited to fight the aberrant type 2 responses typical of food allergy. Certain food antigens can correspondingly favour the development of tolerizing RORγt^+^ cells in the small intestine, specially under Th2 predominant conditions. This immunomodulatory capacity is closely linked to their ability to convert vitamin A into RA, which appears as an essential factor in governing intestinal homeostasis.

## Author contributions

RL: Conceptualization, Funding acquisition, Writing – original draft. EM: Funding acquisition, Writing – review & editing. DL: Writing – review & editing.
